# Gellan Gum Hydrogels Filled Edible Oil Microemulsion for Biomedical Materials: Phase Diagram, Mechanical Behavior, and In Vivo Studies

**DOI:** 10.3390/polym13193281

**Published:** 2021-09-26

**Authors:** Muhammad Zulhelmi Muktar, Muhammad Ameerul Amin Bakar, Khairul Anuar Mat Amin, Laili Che Rose, Wan Iryani Wan Ismail, Mohd Hasmizam Razali, Saiful Izwan Abd Razak, Marc in het Panhuis

**Affiliations:** 1 Faculty of Science and Marine Environment, Universiti Malaysia Terengganu, Kuala Nerus 21030, Terengganu, Malaysia; gsk2761@student.umt.edu.my (M.Z.M.); laili@umt.edu.my (L.C.R.); waniryani@umt.edu.my (W.I.W.I.); mdhasmizam@umt.edu.my (M.H.R.); 2 Pharmaniaga LifeScience Sdn. Bhd., Lot 7, Jalan PPU 3, Taman Perindustrian Puchong Utama, Puchong 47100, Selangor, Malaysia; ameeramin110@gmail.com; 3 BioInspired Device and Tissue Engineering Research Group, Faculty of Engineering, School of Biomedical Engineering and Health Sciences, Universiti Teknologi Malaysia, Skudai 81300, Johor, Malaysia; saifulizwan@utm.my; 4 Faculty Science, Medicine and Health, School of Chemistry and Molecular Bioscience, University of Wollongong, Wollongong, NSW 2500, Australia

**Keywords:** gellan gum, virgin coconut oil, hydrogels, biomaterials, wound dressing

## Abstract

The demand for wound care products, especially advanced and active wound care products is huge. In this study, gellan gum (GG) and virgin coconut oil (VCO) were utilized to develop microemulsion-based hydrogel for wound dressing materials. A ternary phase diagram was constructed to obtain an optimized ratio of VCO, water, and surfactant to produce VCO microemulsion. The VCO microemulsion was incorporated into gellan gum (GG) hydrogel (GVCO) and their chemical interaction, mechanical performance, physical properties, and thermal behavior were examined. The stress-at-break (σ) and Young’s modulus (YM) of GVCO hydrogel films were increased along with thermal behavior with the inclusion of VCO microemulsion. The swelling degree of GVCO hydrogel decreased as the VCO microemulsion increased and the water vapor transmission rate of GVCO hydrogels was comparable to commercial dressing in the range of 332–391 g m^−2^ d^−1^. The qualitative antibacterial activities do not show any inhibition against Gram-negative (*Escherichia coli* and *Klebsiella pneumoniae*) and Gram-positive (*Staphylococcus aureus* and *Bacillus subtilis*) bacteria. In vivo studies on Sprague–Dawley rats show the wound contraction of GVCO hydrogel is best (95 ± 2%) after the 14th day compared to a commercial dressing of Smith and Nephew Opsite post-op waterproof dressing, and this result is supported by the ultrasound images of wound skin and histological evaluation of the wound. The findings suggest that GVCO hydrogel has the potential to be developed as a biomedical material.

## 1. Introduction

There are an overwhelming number of wound dressings available in the market. The high demand for wound dressing is due to the increasing number of wound cases recorded. It is reported that the treatment costs for chronic wounds are substantial and are estimated to account for approximately 1–3% of the total healthcare expenditure in developed countries [1,2]. For example, Wales was estimated to have a chronic wound prevalence of 6% in the year 2012–2013, corresponding to 5.5% of National Health Service expenditure [1], and in the United Kingdom as a whole, the cost associated with wound management was estimated to be GBP 4.5 and GBP 5.1 billion in 2012 [3]. In the United States, it has been reported that over 6.5 million patients with wounds cost the health care system USD 25 billion annually [4]. The point of using wound dressing is to cover the wound from infection and provide appropriate conditions to enhance the healing process of the wounds. However, there is a concern about the usage of hazardous chemicals in the development of wound dressing such as various inorganic nanoparticles. Inappropriate chemicals in wound dressing may lead to other diseases such as skin cancer [5]. The usage of biopolymer and natural products for a better and safer wound dressing is needed. Hence, gellan gum produced by the Gram-negative bacterium of Pseudomonas elodea is chosen due to its biocompatibility and unique properties [6,7].

Gellan gum (GG) is nontoxic, biocompatible, and biodegradable, and the resulting hydrogel is transparent and stable [8]. GG hydrogels are three-dimensional crosslinked polymer networks, a result of transformation from a disordered single coiled structure at high temperature (80 °C) to a double helix and bonded by internal hydrogen bonding between D-glucoronate and D-glucose C residues upon cooling between 30 to 50 °C [9]. In a swollen state these are soft and elastic, resembling the living tissue and exhibiting excellent biocompatibility. The unique properties of hydrogels lead to the wide use of these biomaterials in different fields, including pharmaceutical and biomedical [10,11]. Gellan gum has been proven by the United States Food and Drug Administration (US FDA) and the European Union (EU) to be safely utilized in the food industry. Gellan gum has been studied as matrices to repair and regenerate a wide variety of tissues and organs [12]. The material has also been used as scaffold materials for the application of tissue engineering [6], in the development of wound dressing materials [13], as a vehicle for drug delivery [14], and in eye drops [15]. Gellan gum also has shown good compatibility against various live cells such as mouse fibroblast (L929 cell line) [13], mouse fibroblast cell (3T3) [16], human fetal osteoblast (HFOBs 1.19) [17], human skin fibroblast (CRL2522) [6], and human nasal cartilage [18]. 

Virgin coconut oil (VCO) is essentially colorless, free from rancidity, and endowed with natural antioxidant and vitamin E that prevents the peroxidation reaction. VCO mainly consists of medium-chain triglycerides (MCT) and differs from animal fats that consist of long-chain saturated fatty acids, which is the one main risk factor for cardiac compilation. Based on studies, VCO has been reported to have the potential in promoting the healing process [19]. The oil has been applied in treating wounds in young rats and healed faster by decreasing time for complete epithelization, and results in a significant increase of collagen production, which indicates higher collagen crosslinking. VCO also showed a significant effect in reducing inflammation in acute and chronic inflammation on ethyl phenylpropionate-induced ear edema in rats [20]. A few studies have been reporting the use of biopolymers with essential oil to produce a dressing material [21]. Gellan gum hydrogel films with lavender/tea tree oil showed 98% wound contraction in rats after ten days of treatment and histological images displayed completely healed epidermis [21]. Another study used poly(lactic acid) (PLA) polymer and babassu oil and reported that this material provides a good option for use as wound dressings—films showed a recommended value of the water vapor transmission rate (WVTR), maintained a humid environment above the wound, had good cytotoxicity on normal human keratinocytes (HaCaT), stimulated the keratinocytes migration, and inhibited Pseudomonas aeruginosa growth [22].

Based on the demands to produce a more efficient and safer wound dressing, this study optimizes the ratios of VCO, water, and surfactant by developing a ternary phase diagram and producing a VCO microemulsion. The optimum concentration of VCO microemulsion is selected for incorporation into GG solution and characterized for their chemical interaction, mechanical performance, physical properties, and thermal behavior. Furthermore, the qualitative in vitro antibacterial activities were examined against two Gram-negative (*Escherichia coli* and *Klebsiella pneumoniae*) and two Gram-positive (*Staphylococcus aureus* and *Bacillus subtilis*) bacteria. The in vivo studies were carried out to study the healing properties of the samples on Sprague–Dawley rats, observing the ultrasound images of wound skin and the histological evaluation after the 14th day of post-wound. 

## 2. Materials and Chemicals

### 2.1. Materials

Low-acyl gellan gum (GG, batch no: 5C1574A) was obtained from CP Kelco, Atlanta, GA, USA. Glycerin (product number—G2289), anhydrous calcium chloride, CaCl_2_ (product number—C5670), Tween 80 (product number—P1754), and Triton X-100 (product number—T9284) were obtained from Sigma Aldrich, St. Louis, MO, USA. Virgin coconut oil (product number—VCO0216) was obtained from Phyto Biznet Sdn Bhd, Skudai, Johor, Malaysia. All materials were used as received without further purification.

### 2.2. Construction of Phase Diagrams

Phase diagrams were constructed by mixing two of the components (VCO and water) and titrated using surfactant (Tween 80—Method I or TritonX-100—Method II) as a third component. The surfactant, i.e., Tween 80, was added into the VCO containing distilled water at different ratios (Table 1) and was vortexed (Vortex 3, Eppendorf, Germany) for 3 min. The mixtures were then centrifuged (Minispin Eppendorf, Germany) at 400 rpm for 10 min and later placed in a water bath at room temperature (26 ± 2 °C) for 24 h to record the stability of the mixtures produced. The same procedure was carried out with TritonX-100 (Method II) surfactant at the specific ratios (Table 1). 

### 2.3. Preparation of GVCO Hydrogel

The gellan gum (GG) solutions were prepared by dissolving 2% (*w*/*v*) gellan gum in deionized water (18 MΩ) with 50% (*w*/*w*) glycerin under continuous stirring at 500 rpm for 2 h at 80 °C. VCO microemulsions were prepared by mixing the VCO: Water with Tween 80 surfactant at a specific percentage, as shown in Table 1. For example, to produce the VCO microemulsion of VCO60, 21.38% of VCO was mixed with 14.25% of water with an addition of 64.37% of Tween 80 (Table 1). The same process was carried out for VCO70 and VCO80. To produce GVCO60 hydrogel, 5% (*v*/*v*) of the VCO60 microemulsions were added into the GG solution and stirred for 20 min at 80 °C. The methods were repeated for VCO70 and VCO80 microemulsions. The mixtures were then poured into a Petri dish and allowed to cool at room temperature for 24 h before use for characterization. The GG solution with VCO60 was then known as GVCO60 hydrogels, and the same naming was applied for VCO70 and VCO80 hydrogels. 

### 2.4. Characterisation of the Sample 

#### 2.4.1. Ultraviolet–Visible Spectroscopy

Ultraviolet–visible (UV–Vis) absorption and transmission spectra of solutions and hydrogels were performed using a spectrophotometer (Varian, Cary 50 UV–Vis NIR) with data interval = 5 nm, scan speed = 24,000 nm/min, and wavelength range 500–800 nm. The UV–Vis transmittance was conducted by attaching the hydrogel to the cuvette surface. 

#### 2.4.2. ATR–FTIR Spectroscopy 

ATR–FTIR spectra were collected using a Perkin Elmer Spectrum 100 FTIR spectrophotometer with a PIKE Miracle ATR accessory (single-bounce beam path, 45° incident angle, 16 scans, 4 cm^−1^ resolution). An advanced ATR correction was applied to all spectra in the region from 4000 to 600 cm^−1^.

#### 2.4.3. Mechanical Properties

Mechanical properties of hydrogel films were performed using an Instron Universal Testing Machine (model 3366) with a load capacity and cross-speed according to ASTM standard method D882 (ASTM, 2001). Each sample was cut to 2.0 × 2.0 cm^2^ for stress–strain measurements. The thicknesses of hydrogel films were measured using a handheld micrometer (Mitutoyo Corporation, Mitutoyo, Japan). Stress-at-break (σ), strain-at-break (γ), and Young’s modulus (E) were recorded and a minimum of three independent measurements were obtained per sample. 

#### 2.4.4. Swelling Properties

The swelling properties were determined according to the ASTM Standard Test Methods for One-Dimensional Swell (D4546-08). The swelling was measured by weighing dried films (W_dry_) before immersion into 50 mL phosphate buffer solutions of pH 7.2 at 37 ± 0.5 °C in a water bath. The hydrogels were removed after 24 h, gently wiped with a tissue to expel the surface water, and weighed (W_wet_). Swelling degree (%) was determined according to Equation (1):

Swelling degree (%) = (W_wet_ − W_dry_)/W_dry_ × 100 (1)

where W_dry_ and W_wet_ are the initial weight and final weight, respectively. A minimum of three independent measurements was obtained per sample.

#### 2.4.5. Water Vapor Transmission Rate (WVTR)

The water vapor transmission rate (WVTR) was measured following a modified ASTM International standard method ASTM E96-95. Each hydrogel was fixed on the circular opening of a permeation bottle with an effective transfer area (A) of 1.33 cm^2^. The permeation bottle was placed in the temperature–humidity chamber at 37 °C and 50 ± 5% relative humidity. The equilibrium moisture penetration was determined by weighing the bottles at 0 and 24 h. The WVTR was calculated according to Equation (2):

WVTR = (m/∆T)/A.(2)

where m/∆T is the amount of water gain per unit time of transfer and A is the area exposed to water transfer (m^2^).

#### 2.4.6. Thermogravimetric Analysis

Thermogravimetric analyses were performed on a Pyris 6, Perkin-Elmer-TGA6. Hydrogel samples were analyzed in platinum pans at a heating rate of 10 °C/min to 275 °C in an atmosphere of N_2_ atmosphere at a flow rate of 50 mL/min. The sample used was approximately 10 mg.

#### 2.4.7. Scanning Electron Microscopy (SEM)

The cross-section morphology of the hydrogels was acquired using a JOEL JSM 6360 LA electron microscope. Scanning electron microscopy (SEM) images of cross-sections were obtained by freeze-dried technique. Samples were freeze-dried in liquid nitrogen (−160 °C) and fractured at −150 °C at the middle of hydrogels. It was then coated with Auto Fine Coats (JFC-1600) before microscopic observation.

### 2.5. Antibacterial Study

#### 2.5.1. Culture Conditions 

Gram-positive (*Staphylococcus aureus* and *Bacillus subtilis*) and Gram-negative (*Escherichia coli* and *Klebsiella Pneumoniae*) microbes were used for antibacterial assays. The standard growth medium Mueller–Hinton (MH, Difco, Sparks, MD, USA) agar was used for the growth of both bacterial types and prepared by sterilization using an autoclave (15 min, 120 °C). These two species of microbes were evenly spread on the solid MH agar and incubated aerobically at 37 °C for 24 h. The bacterial suspension was measured by using Spectrophotometer BiomerieuxDensicheck Plus at 600 nm to obtain optical density at 0.5.

#### 2.5.2. Qualitative Study

The Gram-positive and Gram-negative bacteria suspensions were evenly spread on the solid MH agar in the sterile Petri plates. Using a sterile cotton swab, both bacteria were swabbed over the surface of the agar plates and dried in a laminar flow air chamber. The hydrogel sample was gently pressed on the agar with bacteria and incubated at 37 °C for 24 h in triplicates. The presence of any clear zone around the disc on the MH agar was recorded as an indication of inhibition against the bacteria.

### 2.6. In Vivo Studies

#### 2.6.1. In Vivo Wound Healing Experiments

##### Animals

In this study, a total number of 20 six-week-old female Sprague–Dawley rats with a range of body weight from 200–250 g were used. They were randomly divided into three experimental groups of five rats each. The animals were acclimatized to the laboratory conditions for one week before the onset of the experiment. All rats were individually caged with a 12-h light/dark cycle, given adequate commercial pellets and water ad libitum throughout the study. All animal experiments were carried out under protocols approved by the Animal Ethics Committee (AEC)—UMT/JKEPHMK/2020/48, Universiti Malaysia Terengganu.

##### Establishment of Wound Skin

Rats were anaesthetized using an intraperitoneal (i.p.) injection of ketamine (90 mg/kg) and xylazine (10 mg/kg). The dorsal skin was prepared by removing the hair with a razor blade and the surgical area was disinfected with 70% ethanol. Since the shaving procedure produced marked edema of the skin, the prepared rats were left for twenty-four hours before the wound was inflicted. After the rats were anesthetized with a combination of ketamine and xylazine via i.p., a full-thickness wound was created by using an 8-mm sterile skin biopsy punch. Each rat received two full-thickness wounds at their back dorsal. 

##### Treatment of Hydrogel

All wounds in the treatment groups were dressed with GG and GVCO80 hydrogels, and followed by Opsite post-op waterproof film dressing (Smith and Nephew, Hull, England) as the secondary dressing. The dressings were then held in place with gauze to give mechanical protection to the dressing. The changing of dressing was done every 3 days to minimize the infection to the wound site. The Opsite film dressing acted as a positive control for comparison to the other treatments. The treated wound with GG dressing was considered a negative control. The rat’s wound was photographed using a 13.1-megapixel Sony camera for evaluation of wound closure with the actual measurement.

##### Macroscopic Observation of Wound

The wound measurement of the size taken at the time of biopsy was used to calculate the percent of wound contraction using equation:

(3) $$\;\%\;\mathrm{wound}\;\mathrm{contraction}\;=\frac{W_{0}\;-\;W_{t}}{W_{0}\;}\times 100$$

where W_0_ is the original wound area and W_t_ is the wound area on the selected day after the biopsy. The measurements of wound size were taken on days 2, 4, 7, 11, and 14 consecutively throughout the study. The wound area was measured by placing the 1 mm^2^ graph over the wound pictures. The squares were counted, and the area was recorded. The wound area was accessed by the same blinded observer.

#### 2.6.2. Ultrasound Imaging

The wound area also was analyzed by using real-time high-resolution 20 MHz ultrasound imaging equipment (Dermalab Combo, Cortex, Denmark) skin analyzer to produce images representing the cross-section of the wound skin. A standard echographic gel was applied and used as a medium between the probe and wound skin surface. The images produced were recorded. 

#### 2.6.3. Histological Examination

Rats were euthanized on day 14 and skin samples that contained the wound area were taken for histological study. The skin samples were fixed with 10% buffered formalin for 24 h. The samples were embedded in paraffin and cut into 6 mm-thick sections for the middle part of the wounds. The sections were subsequently stained with hematoxylin and eosin (H & E) staining procedure. The H & E slides were visualized using a light microscope at 20x magnification. 

##### Statistical Analysis

All data are presented as the mean ± standard deviation (SD). The data were processed by two-way ANOVA using statistical software analysis SPSS (version 20). The *p*-value < 0.05 was considered statistically significant.

## 3. Results and Discussion

### 3.1. Formulation of Stabilizing VCO Microemulsion

**Figure 1:**
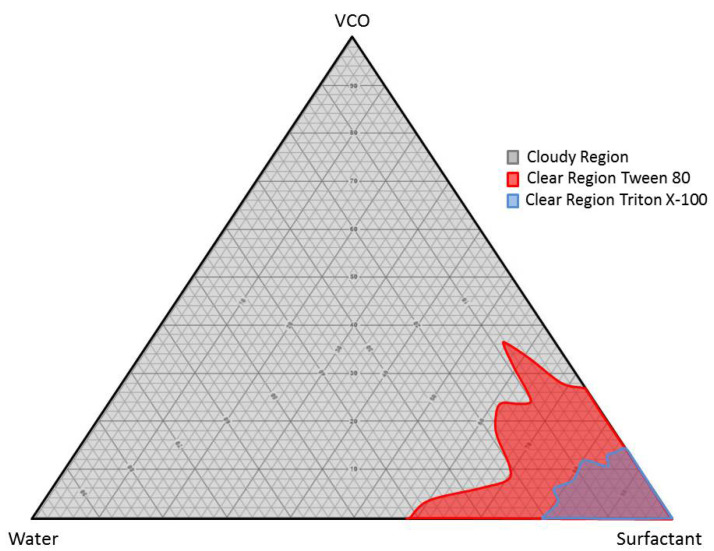
The ternary phase diagram of VCO microemulsion by using Tween 80 and TritonX-100 surfactants.

In general, water and oil were separated due to their high interfacial tension. Water is immiscible with virgin coconut oil (VCO) due to its high interfacial tension, which is typical for oils with water. The interfacial tension was reduced by introducing surfactant, which allowed the microemulsion polymerization process to take place. Nonionic surfactants, Triton X-100 and Tween 80, were used in preparing a stabilized VCO microemulsion through a ternary phase diagram. The optimum ratio from the ternary phase diagram was chosen in preparing the gellan gum–virgin coconut oil hydrogels (GVCO). The microemulsion has a characteristic of a clear solution, stable at room temperature and forming a one layer solution. The pseudo ternary phase diagrams consisting of virgin coconut oil microemulsion, distilled water, and surfactant with different hydrophilic–lipophilic balance (HLB) values were constructed using the surfactant titration method. Nine ratios of VCO: Water were constructed, i.e., 90:10, 80:20, 70:30, 60:40, 50:50, 40:60, 30:70, 20:80, and 10:90 (*w*/*w*). Two types of nonionic surfactants were used, which were Tween 80 and Triton X-100 (Table 1). The mixture was observed using visual inspection after each addition of surfactant to the VCO and water. The samples were identified as microemulsions when they appeared transparent, and the liquid easily flowed. The results were plotted on a triangular graph as a ternary phase diagram, as shown in Figure 1.

The ternary phase diagram shows that different amounts of VCO, water, and Tween 80 can produce a stable oil-in-water microemulsion. The ratio of VCO: Water required a different amount of Tween 80 in the range from 55.59% to 69.50% to achieve a stable phase of microemulsions (Table 1). The ratios of 80:20 needed 55.59% of Tween 80 compared to the ratio of 70:30 and 60:40, which only needed 65.73% and 64.37%, respectively (Table 1). The volume of Triton X-100 required to produce a stable formulation in VCO: Water was higher than in Tween 80. The Triton X-100 amount ranged from 78.09% to 86.07% to produce stable microemulsions (Table 1). The amount of Triton X-100 was 83.24%, 83.63%, and 79.62% for ratios 80:20, 70:30, and 60:40, respectively. The formulation of VCO:Water: Tween 80 microemulsion was adopted due to the large area of microemulsion stability in the ternary phase diagram and lower amount of surfactant needed to form a stable mixture, compared to Triton X-100. Moreover, Tweens were reported to have been used widely in food, cosmetics, and pharmaceutical application due to minimal toxicity and low cost [23,24]. 

### 3.2. Physical Properties of the Hydrogels

**Figure 2:**
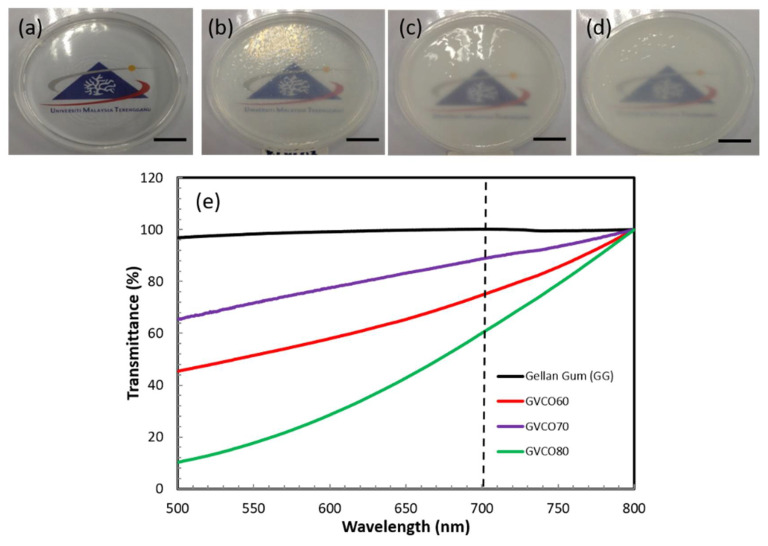
The physical appearance of (**a**) GG hydrogel, (**b**) GVCO60 (**c**) GVCO70, (**d**) GVCO80 hydrogels, and (**e**) transmittance of GG and GG–VCO hydrogels.

The addition of VCO microemulsion to the gellan gum does not change the physical appearance of transparent free-standing GG hydrogel (Figure 2). The GVCO hydrogels at every concentration were transparent but becoming cloudy due to the increased amount of VCO microemulsion and supported by the transmittance result (Figure 2e). The transmittance for pure GG hydrogels was ≈ 99% compared to GVCO60 ≈ 85%, GVCO70 ≈ 70%, and GVCO80 ≈ 65% at λ = 700 nm. The transparent behavior of the GVCO hydrogel gives an advantage for the materials to be used as wound dressing material, and the healing process could be monitored with ease.

### 3.3. Fourier Transform Infrared Spectroscopy (ATR–FTIR)

**Figure 3:**
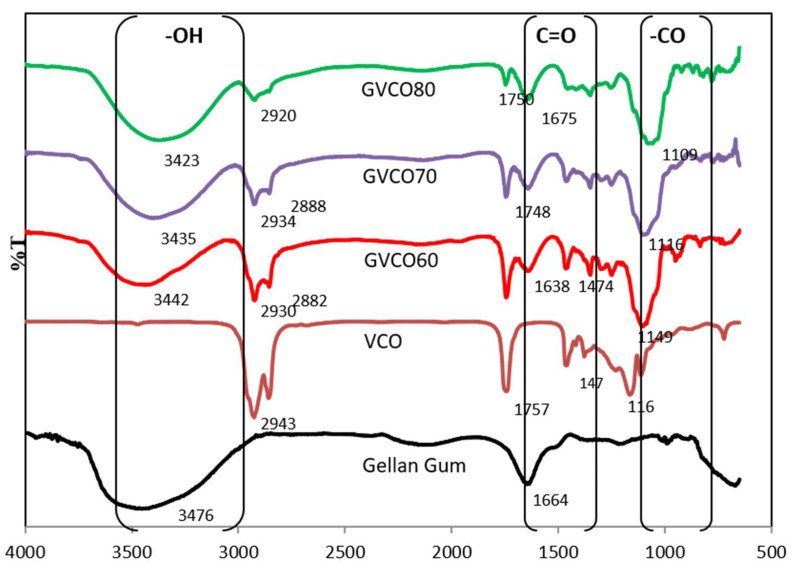
ATR spectra of pure GG hydrogels, VCO, and GVCO hydrogels at different concentrations.

ATR–FTIR spectra of the GVCO hydrogels confirm the presence of characteristic peaks of GG and VCO (Figure 3). The peaks at 2941 and 2895 cm^−1^ are related to the saturated alkyl and the carbonyl groups of the fatty acids in VCO, respectively [25]. Peaks appearing at 1758, 1470, and 1419 cm^−1^ were from the stretching of carboxylic (C=O), bending of methylene (CH_2_), and bending of methyl (CH_3_) of VCO. The 1182 and 1142 cm^−1^ peaks resulted from the stretching of ester C-O [26]. 

GG has a broad band at 3476 cm^−1^, which is a typical region for stretching of the O-H group (3000–3500 cm^−1^) [27]. A new distinguishing peak observed at 1638–1675 cm^−1^ in GVCO hydrogels correlates to the shifting of -C-H (-CH_2_) bending scissoring of VCO and C=C due to the appearance of phenolic content and stretching of that aromatic ring [28]. The stretching alkyl signal in VCO microemulsion at 2943 cm^−1^ shifted to 2930–2920 cm^−1^ in GVCO hydrogels confirming the hydrogen bonding interaction in GVCO blends [28]. 

A significant change of enlargement peak of GVCO hydrogel can also be observed around 1149–1109 cm^−1^, due to an increased stretching vibration of esters group of gellan gum and VCO. The ATR–FTIR spectra of GVCO show all prominent peaks of VCO with significant changes in variation and shifting of transmittance intensities, indicating that GG and VCO microemulsion interact to produce a stable network hydrogel. 

### 3.4. Mechanical Performances

**Figure 4:**
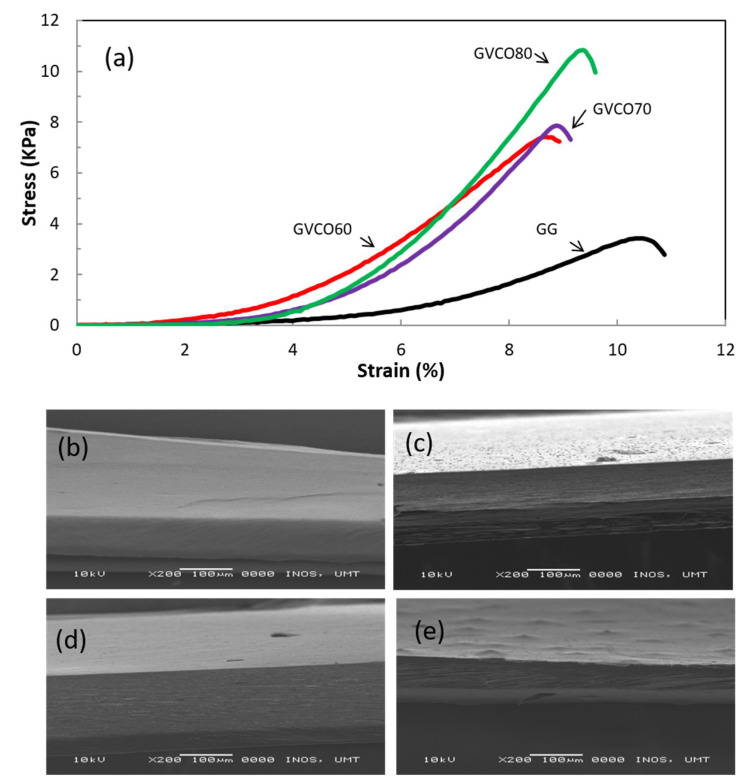
(**a**) Typical stress–strain curves of GG and GVCO hydrogels, (**b**–**e**) scanning electron microscopy images of the cross-sectional area of (**b**) pure gellan gum (GG) hydrogels, (**c**) GVCO60, (**d**) GVCO70, and (**e**) GVCO80 hydrogels.

Mechanical performance is crucial to evaluate and understand the strength of the materials. Table 2 shows the tensile properties of GVCO hydrogels at the different ratios on the stress-at-break (σ), strain-at-break (γ), and Young’s modulus (YM). Free-standing gellan gum (GG) hydrogel was brittle and almost impossible for use in biomedical applications such as a wound dressing material. To overcome this problem, VCO microemulsions were incorporated into GG as a plasticizer and to improve the flexibility of the materials. The incorporation of VCO microemulsion into GG caused the hydrogels to become durable, flexible, and easy to handle due to an increase in stress-at-break (σ) and Young’s modulus (YM), but decreased slightly for strain-at-break (γ) values (Figure 4a). The σ and YM values of GG hydrogel were 4 ± 0.2 and 85 ± 7 kPa, respectively, and the addition of VCO microemulsion (GVCO80) increased both values to 11 ± 0.3 and 259 ± 7 kPa, respectively (Table 2). Although the γ value of the GVCO60 decreased to 7.9 ± 0.2% from 10.7 ± 0.4% (GG hydrogel), the addition of higher content of VCO microemulsion resulted in the γ value increasing to 9.3 ± 0.4% compared to GVCO60 hydrogel films. Thus, the plasticizer effect of the VCO microemulsion in GCVO hydrogel films was proven. 

The mechanism of GG hydrogel formation is closely related to the conformational transition from the coil to helix structures [13]. Normally, GG hydrogels are produced by physical crosslinking methods induced by temperature variation or by the presence of divalent cations [29]. Normal hydrogels are usually spontaneously formed by weak secondary forces such as hydrogen bonding, van der Waals interactions, and ionic bonding [30]. In an aqueous solution at high temperatures (≈60 °C), the gellan gum chain is in a disordered single-coil state. Upon cooling from 70 to 30 °C, the gellan solution promotes the formation of double helices stabilized by internal hydrogen bonding [31]. This facilitates the tight packaging of gellan gum chains, resulting in fragile hydrogels. However, the addition of VCO microemulsion that contains lauric acid (C12 ≈ 78%) and a hydroxyl group is responsible for promoting the formation of hydrogen bonds between gellan gum and VCO microemulsion [28]. This bonding replaces the hydrogen bonds between gellan gum chains and thus decreases the intermolecular bonds along polymer chains and improves strain-at-break [6] of the hydrogels regarding the content of VCO microemulsion. The bonding is expected to increase the stress-at-break values and give more strength to the hydrogels. Another factor that was contributing to improving flexibility of the GVCO hydrogels could be due the polymerization process. The packed and solid structure of GG and GVCO hydrogels was observed in the cross-section of hydrogels. This shows that VCO microemulsion and GG were homogenous, and no separation within the mixture occurred. 

### 3.5. Swelling Ratio, and Water Vapor Transmission Rate (WVTR)

The swelling degree of GVCO hydrogels is summarized in Table 2. The standard buffer solution with pH ≈ 7.2 was used to mimic human body fluid. At pH ≈ 7.2, the number of ionic groups is highest in the solution. The GVCO60 absorbed the maximum swelling rates ≈ 12 ± 4, which is an increment of four-fold more than GG hydrogel. The increase of swelling is expected due to the reaction of –OH groups of VCO with the –OH and –COOH of the polymeric chains [32]. These functional groups can relieve the entanglement of polymeric chains, thus weakening the hydrogen bonding among the hydrophilic groups. This decreases the degree of physical crosslinking and therefore improves the water uptake of the hydrogels. However, the swelling degree for GVCO70 and GVCO80 hydrogels decreased slightly upon adding a higher concentration of VCO microemulsion, 9 ± 1.5% and 6 ± 1.5%, respectively. The other reason could be due to the hydrophobicity property of VCO itself; the greater the amount of VCO, the greater water resistance of the hydrogels expected.

The water vapor transmission rate (WVTR) of control and GVCO hydrogels are in the range of 332–1547 g m^−2^ d^−1^ (Table 2). The value decreased upon the addition of VCO microemulsion that can be expected due to tight packaging of VCO and GG blends, which then interrupted the water vapor transmission rate. The tight packaging of GG and VCO microemulsion resulted in reducing the surface tension and increasing the stability of water retention in GVCO hydrogels. However, these values remain within the range of WVTR values (90–2893 g m^−2^ d^−1^), as reported for eight commercially available synthetic wound dressings [33].

### 3.6. Thermal Analysis 

**Figure 5:**
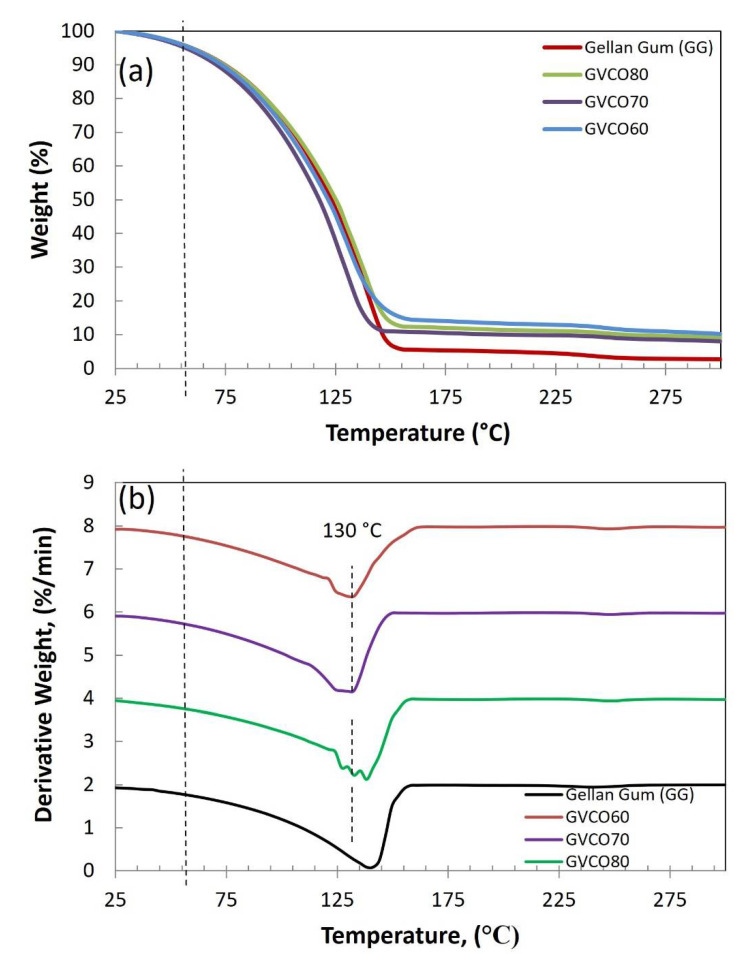
(**a**) Thermogravimetric thermograms and (**b**) derivative thermograms of GG and GVCO hydrogels at different concentrations.

Thermogravimetric analysis (TGA) was conducted to study the thermal stability of GG and GVCO hydrogels. The thermogram and derivative thermogram of GG and GVCO hydrogels are presented in Figure 5. The degradation below T ≤ 100 °C, which occurred for GG hydrogels, is common due to evaporation of moisture in the hydrogels [6]. The degradation of GG and GVCO hydrogels shows a similar trend, in which the degradation occurred at the onset temperature ≈ 54 °C, and the offset temperature at ≈ 160 °C (Table 3). However, there are differences in the peak of GVCO hydrogels at ≈ 130 °C. The GVCO80 hydrogels (containing the highest concentration of VCO) show three small peaks at ≈ 130 °C and are assumed due to a few degradation processes of polymer materials. The GVCO60 and GVCO70 hydrogels have two peaks (at ≈ 130 °C), and a broader peak at the center (Figure 5). The appearance of these small peaks at ≈ 130 °C shows that the addition of VCO exhibits a better outcome in increasing the thermal stability of hydrogels due to a certain degree of interaction between GG and VCO. The weight loss of the GVCO hydrogels is further supported the thermal behavior of the hydrogels, in which the weight loss of GVCO60, GVCO70, and GVCO80 decreased to 86%, 89%, and 88%, respectively, compared to GG hydrogel at 95% (Table 3). Decreased weight loss reflects the increases in the thermal behavior of a material [34]. Similar phenomena have been observed for other oil-based materials [35,36]. 

### 3.7. Antibacterial Studies 

**Figure 6:**
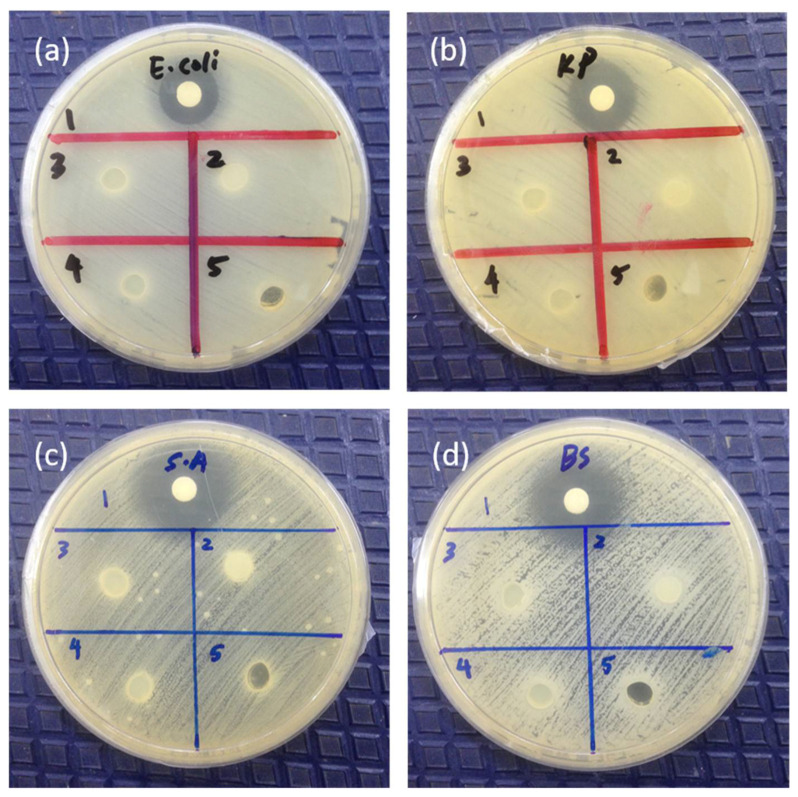
(**a**) *Escherichia coli* (*E. coli*), (**b**) *Klebsiella pneumoniae* (KP), (**c**) *Staphylococcus aureus* (S.A), and (**d**) *Bacillus subtilis* (BS) shown in the figure. Note: The number refers to samples: (1) penicillin (positive control), (2) GVCO80, (3) GVCO70, (4) GVCO60, and (5) gellan gum (negative control).

GG is well known for its behavior to promote cell growth. For that reason, the result confirming that the control (GG hydrogel) shows no antibacterial activities examined through a qualitative method (inhibition zones) after incubating for 24 h against Gram-negative (*Escherichia coli* and *Klebsiella pneumoniae*) and Gram-positive (*Staphylococcus aureus* and *Bacillus subtilis*) bacteria (Figure 6a–d). The addition of VCO microemulsion (GVCO60, GVCO70, and GVCO80) in gellan gum hydrogels, however, results in no clear zone of inhibition around the samples against all bacteria (Figure 6). It has been elaborated that the VCO does not possess antibacterial activity on its own, but rather is induced by its free fatty acids, particularly lauric acid (C12), and to a small extent by capric acid (C10) and caprylic acid (C8) [37]. In other words, the VCO must be metabolized to release those components and exert its antimicrobial effects [38]. Another mechanism proposed for the antibacterial effect of VCO is the lipolyze process with lipase and water to form monoglyceride, for which the structure consists of glyceride molecules attached to either sn-1 or sn-2 position of the glycerol [39]. The derivative of monoglycerides, known as monolaurin or monoester of the lauric acid is the most effective in inhibiting the microorganism by disrupting the cell membrane and the cytoplasm of cells [40].

In this particular study, the GVCO hydrogels do not show antibacterial properties against Gram-negative (*Escherichia coli* and *Klebsiella pneumoniae*) and Gram-positive (*Staphylococcus aureus* and *Bacillus subtilis*) bacteria, maybe due to lesser contact of VCO with the agar, which reduces the chances of the latter to diffuse into the agar. As discussed earlier, the antibacterial activities of VCO are triggered by the monolaurin of lactic acid, which depends on the concentration of VCO. Higher content of VCO may contain a higher content of monoglyceride to transform to monolaurin derivatives and thus exert the antimicrobial effects. However, our result is contrary to a few studies reported previously [36,41]. Although these studies examined the film on the agar spread with Gram-negative and Gram-positive bacteria, which has the lesser contact of essential oil to the agar, but their observation shows promising antibacterial activities. Lee and coworkers reported the antibacterial activities of hydroxypropyl methylcellulose/ oregano essential oil nanoemulsion (HPMC/ORNE) composite films against Gram-positive (*S. aureus*, *B. cereus*, and *L. monocytogenes*) and Gram-negative bacteria (*E. coli*, *S. typhimurium*, *P. aeruginosa*, and *V. parahaemolyticus*) [41]. Their results show that the diameters of the inhibition zones increased significantly (*p* < 0.05) with increasing oregano essential oil concentration, with films containing over 5.0% (*v*/*v*) ORNE showing antibacterial activity against all tested strains, particularly against *S. typhimurium*. The authors claimed the antibacterial activity was attributed to the concentration of its most active compound, namely carvacrol, and other phenolics such as thymol and p-cymene, which can act synergistically, and later disrupting the outer membrane of bacteria, resulting in increased permeability and loss of ions, ATP, and other cytoplasm contents, thus inhibiting bacterial growth [41]. Another study reported by Fangfang and coworkers also shows the antibacterial activity of virgin coconut oil (VCO) incorporated into potato starch-based biodegradable (PSBB) films [36]. According to the authors, the antibacterial effect of films with different VCO concentrations is due to metabolization of the lauric acid in VCO and produce mononucleotides, which have antibacterial properties against *E. coli* and *S. aureus* [36]. Further study should be carried out to understand the possible interaction of VCO with bacteria on the agar at higher concentrations. 

### 3.8. In Vivo Studies

For in vivo studies, one sample of GVCO hydrogel was chosen, i.e., GVCO80 hydrogel. This sample was chosen due to the optimum properties shown by the latter compared to GVCO60 and GVCO70 hydrogels. The GVCO80 hydrogels show a transparent appearance, optimum mechanical properties, and thermal behavior, together with acceptable swelling ratio and WVTR values. Table 4 shows the wound contraction of the GG and GVCO80 hydrogels compared to the commercial product, Opsite dressing. For the first 7 days, the Opsite dressing shows a significant acceleration of healing at 49 ± 11%, followed by GVCO80 hydrogel at 46 ± 7%. The healing process is further enhanced on day 11 when the GVCO80 surpassed the wound closure, compared Opsite dressing, and achieved 95 ± 2% on day 14. The Opsite dressing completed the closure at 93 ± 4 % on day 14. The GG hydrogel shows the lowest percentage of wound closure, 91 ± 4%, compared to other treatments. 

**Figure 7:**
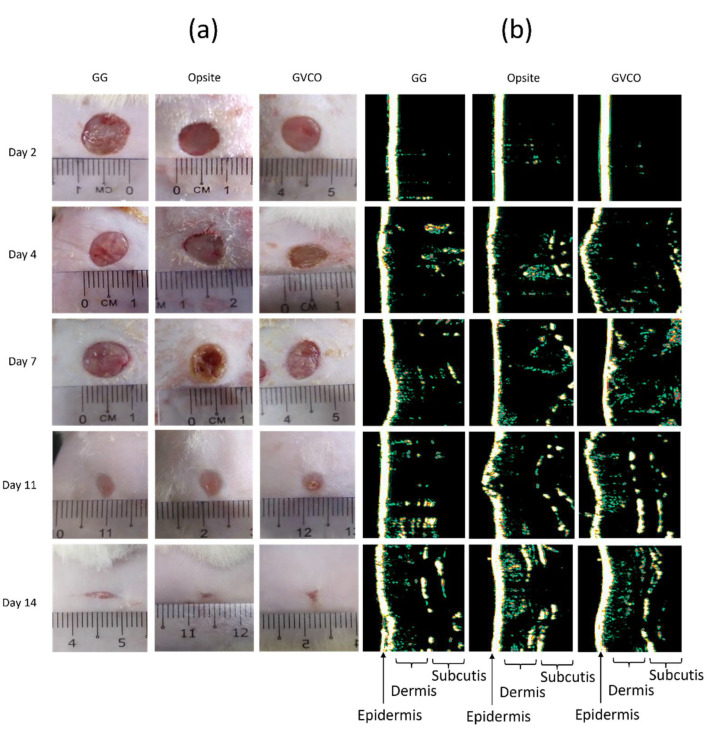
(**a**) Wound healing studies and (**b**) typical ultrasound images of wound skin on gellan gum (GG), Opsite, and GVCO80 hydrogels on days 2, 4, 7, 11, and 14.

In this study, no skin irritation is observed for all treatments. This indicated that the GG is a safe material and a good candidate in biomedical applications [29]. The wound healing process was monitored on days 2, 4, 7, 11, and 14 by capturing images of each animal (Figure 7a). The results are in agreement with the wound contraction (Table 4) in which the GVCO80 hydrogel accelerated the wound closure and the wound gradually disappeared through time. The ultrasound images of the thickness growing on the wound skin are shown in Figure 7b. It shows that the skin formation of a wound treated with GVCO80 hydrogel exhibited the optimum recovery compared to other samples on days 2, 4, 7, 11, and 14. The intensity of white/yellowish/green color indicates the good formation of epidermis, dermis, and subcutis of GVCO80 hydrogel and followed by Opsite. The dermis layer was characterized by varying intensities (different colors) present on the wound while the subcutis layer referred to the low-intensity areas due to the homogenous composition. Subcutis areas are described as the black areas, which are referred to the homogeny structure, such as fat, water, and blood. The GG hydrogel as control showed less intensity among the others. The epidermal regeneration was observed in all experimental groups after the 14th day of treatment.

**Figure 8:**
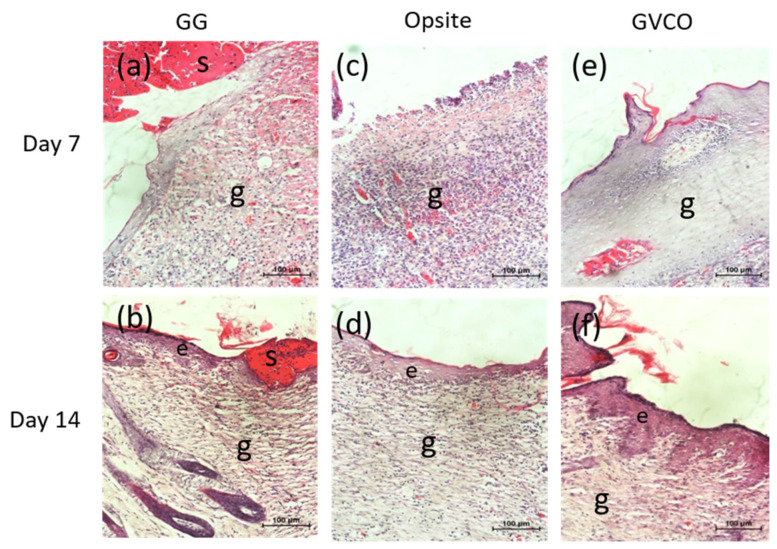
Representative images of the histological evaluation section on day 7 and 14 post-wound, stained with H & E (**a**,**b**) GG, (**c**,**d**) Opsite, and (**e**,**f**) GVCO80 hydrogel. Note: e = epidermis, g = granulation, s= scab; 20x magnification. The bar on the micrograph represents 100 µm.

Hematoxylin and eosin (H & E) staining was performed to evaluate the quality of the wound tissue. Histological evaluation results show that the GVCO80 hydrogel resulted in better re-epithelization as compared to other samples (Figure 8). For Opsite and GVCO80 hydrogel, the formation of epithelial growth was observed and the number of inflammatory cells reduced. Meanwhile, in the control group (GG hydrogel), the new epithelium was noted to regenerate and a little scab was spotted and the necrotic tissue was found under defect. The skin treated with GVCO80 hydrogel and Opsite presented a better result than the control.

The moist environment characteristic of hydrogel is most suited for re-epithelization and enhancing wound healing mechanism on the skin [42]. For a long time, VCO has been a well-known and powerful substance for treating wounds, mainly due to its anti-bacterial, anti-inflammatory, and anti-oxidant properties [43]. An in vivo study conducted by Soliman and coworkers evaluated the effects of topical application of VCO on wound healing in diabetes-induced Sprague–Dawley rats [44]. They found that the wound closure rate in the VCO group was higher on all days compared to diabetic nontreated rats, and VCO was found to be better than silver sulfadiazine cream in the healing of diabetic wounds via promoting re-epithelialization and collagen synthesis, as well as increasing WCR and total protein content. A few other studies reported the effectiveness of pure VCO (liquid form) in promoting the healing process [45,46,47]. The wound healing potency of fermented virgin coconut oil was also verified against human umbilical vein endothelial (HUVEC), fibroblast (CCD-18), and retinal ganglion (RGC-5) cells, as well as a wound excision model in Sprague–Dawley rats [48]. Their finding shows that the expression of phospho-VEGFR2 (vascular endothelial growth factor receptor 2) in HUVECs was detected by Western blot; rats in the VCO group had significantly smaller wound size, higher wound healing percentage, and shorter wound closure time when compared with a control group. Their study also confirmed that a high angiogenic and wound healing potency of VCO contributed to the regulation of the VEGF signing pathway [48]. These past studies show that the VCO significantly affected the healing of the wounds and show promising results to be applied in biomedical applications. With this noted, our study shows that the inclusion of VCO into a biopolymer does not stop the effectiveness of the oil to promote and enhance the healing process. Similar results are obtained, thus indicating this GVCO hydrogel as a promising candidate to be used as dressing materials.

## 4. Conclusions

This study successfully prepared gellan gum (GG) hydrogel incorporated with virgin coconut oil (VCO) microemulsion with the addition of surfactants, i.e., Tween 80 or TritonX-100. A ternary phase diagram was constructed to obtain an optimized ratio of VCO, water, and Tween 80, which was chosen due to optimum concentration to produce a stable VCO microemulsion. The VCO microemulsion was incorporated into gellan gum (GG) hydrogel (GVCO). Its chemical interaction, mechanical performance, physical properties, and thermal behavior were examined. The stress-at-break (σ) and Young’s modulus (YM) of GVCO hydrogel films increased, together with thermal behavior, following the inclusion of VCO microemulsion. The swelling value of GVCO hydrogel decreased as the VCO microemulsion increased and the water vapor transmission rate of GVCO hydrogels was comparable to commercial dressing in the range of 332–391 g m^−2^ d^−1^. Meanwhile, the in vitro qualitative antibacterial study of GVCO hydrogels against Gram-negative (*Escherichia coli* and *Klebsiella pneumoniae*) and Gram-positive (*Staphylococcus aureus* and *Bacillus subtilis*) bacteria showed that VCO possesses a weak antibacterial effect. In vivo studies on Sprague–Dawley rats show the wound contraction of GVCO80 hydrogel is the best (95 ± 2%) after the 14th day compared to Smith & Nephew Opsite post-op waterproof dressing at 93 ± 4%, and supported by the ultrasound images of wound skin and histological evaluation on the wound. This study demonstrated that the GVCO hydrogels had the potential to be used as wound dressing materials.

## Figures and Tables

**Table 1:** Ratios of VCO, water, and surfactants (Tween 80—Method I and TritonX-100—Method II) to develop a ternary phase diagram and produce a stable VCO microemulsion.

	Method I				Method II			
Ratio VCO: Water	VCO (%)	Water (%)	Tween 80 (%)	Total (%)	VCO (%)	Water (%)	TritonX-10 (%)	Total (%)
90:10	27.45	3.06	69.49	100	12.54	1.39	86.07	100
80:20	35.52	8.88	55.59	100	13.41	3.35	83.24	100
70:30	23.98	10.29	65.73	100	11.46	4.91	83.63	100
60:40	21.38	14.25	64.37	100	12.23	8.15	79.62	100
50:50	16.43	16.43	67.14	100	9.66	9.66	80.68	100
40:60	17.27	25.91	56.84	100	7.82	11.74	80.44	100
30:70	9.15	21.35	69.50	100	6.57	15.34	78.09	100
20:80	6.96	27.86	65.18	100	3.88	15.55	80.57	100
10:90	4.03	36.23	59.74	100	2.29	16.80	80.91	100

**Table 2:** Summary of the stress-at-break (σ), strain-at-break (γ), Young’s modulus (YM), swelling degree, and water vapor transmission rate (WVTR) of gellan gum (GG) and GVCO hydrogels at different ratios. Control for WVTR is a test without hydrogel film.

	Stress (σ) (kPa)	Strain (γ) (%)	Modulus (YM) (kPa)	Swelling Degree (%)	WVTR (g m^−2^ d^−1^)
Control	-	-	-	-	1547 ± 32
GG	4 ± 0.2	10.7 ± 0.4	85 ± 7	3 ± 0.6	964 ± 47
GVCO60	6 ± 0.1	7.9 ± 0.2	134 ± 6	12 ± 4.0	391 ± 13
GVCO70	7 ± 0.4	8.5 ± 0.2	175 ± 8	9 ± 1.5	344 ± 23
GVCO80	11 ± 0.3	9.3 ± 0.4	259 ± 7	6 ± 1.5	332 ± 11

**Table 3:** Thermal gravimetric properties of gellan gum (GG) and GVCO hydrogels.

Sample	T_o_ (°C)	Temperature Offset (°C)	Weight Loss (%)
GG	54	157	95
GVCO60	48	165	86
GVCO70	53	155	89
GVCO80	50	157	88

**Table 4:** The percentage of wound contraction of experiment groups on days 2, 4, 7, 11, and 14; the means of two replicates where Opsite acts as a control; * (*p* < 0.05 compare with the control group).

Day/Treatment	2^nd^	4^th^	7^th^	11^th^	14^th^
	Percentage (%) of Wound Closure (Mean ± SD)				
GG	9 ± 5	25 ± 7	35 ± 2	81 ± 6	91 ± 4
Opsite	25 ± 3 *	31 ± 3	49 ± 11	88 ± 5	93 ± 4
GVCO80	11 ± 3	28 ± 2	46 ± 7	89 ± 3	95 ± 2

## Data Availability

The data presented in this study are available on request from the corresponding author.
